# Abnormal Upregulation of GPR17 Receptor Contributes to Oligodendrocyte Dysfunction in SOD1 G93A Mice

**DOI:** 10.3390/ijms21072395

**Published:** 2020-03-31

**Authors:** Elisabetta Bonfanti, Tiziana Bonifacino, Stefano Raffaele, Marco Milanese, Erica Morgante, Giambattista Bonanno, Maria P. Abbracchio, Marta Fumagalli

**Affiliations:** 1 Department of Pharmacological and Biomolecular Sciences, Università degli Studi di Milano, 20133 Milan, Italy; 2 Department of Pharmacy and Center of Excellence for Biomedical Research (CEBR), University of Genoa, 16132 Genoa, Italy; 3 IRCCS Ospedale Policlinico San Martino, 16132 Genoa, Italy

**Keywords:** G protein-coupled receptor 17 (GPR17), amyotrophic lateral sclerosis (ALS), SOD1^G93A^ ALS mouse model, oligodendrocytes, montelukast

## Abstract

Amyotrophic lateral sclerosis (ALS) is a neurodegenerative disease characterized by progressive loss of motor neurons (MN). Importantly, MN degeneration is intimately linked to oligodendrocyte dysfunction and impaired capacity of oligodendrocyte precursor cells (OPCs) to regenerate the myelin sheath enwrapping and protecting neuronal axons. Thus, improving OPC reparative abilities represents an innovative approach to counteract MN loss. A pivotal regulator of OPC maturation is the P2Y-like G protein-coupled receptor 17 (GPR17), whose role in ALS has never been investigated. In other models of neurodegeneration, an abnormal increase of GPR17 has been invariably associated to myelin defects and its pharmacological manipulation succeeded in restoring endogenous remyelination. Here, we analyzed GPR17 alterations in the SOD1^G93A^ ALS mouse model and assessed in vitro whether this receptor could be targeted to correct oligodendrocyte alterations. Western-blot and immunohistochemical analyses showed that GPR17 protein levels are significantly increased in spinal cord of ALS mice at pre-symptomatic stage; this alteration is exacerbated at late symptomatic phases. Concomitantly, mature oligodendrocytes degenerate and are not successfully replaced. Moreover, OPCs isolated from spinal cord of SOD1^G93A^ mice display defective differentiation compared to control cells, which is rescued by treatment with the GPR17 antagonist montelukast. These data open novel therapeutic perspectives for ALS management.

## 1. Introduction

Amyotrophic lateral sclerosis (ALS) is the most common adult onset motor neuron (MN) disorder involving MN degeneration and causing serious and irreversible muscle weakness and atrophy, with death of patients within 3–5 years from diagnosis [1]. Approximately 10% of patients have a familial form of this disease and among these, about 20% of ALS cases are caused by mutations in the gene encoding for superoxide dismutase 1 enzyme (SOD1) [2].

For a long time, ALS has been only considered a disease of the grey matter (GM) involving MN degeneration; however, this ‘neuron-centric’ view has been increasingly challenged over the last years. Alterations in the white matter (WM) structure have been reported to be more pronounced compared with those in MN structures [3] and have been described also in the perforant path area of ALS patients [4], likely contributing to typical ALS symptoms which include motor and neurological deficits and cognitive decline. Globally, these observations have laid the foundation to consider ALS as a non-cell autonomous disease [5]. Accordingly, a growing amount of evidence has shown that mutant SOD1 expression in non-neuronal cells, mainly astrocytes and microglia, has deleterious effects that contribute to neuronal death [6,7,8,9].

Recent studies have demonstrated that also oligodendrocytes, the highly specialized post-mitotic cells that provide trophic and structural support to neurons in both GM and WM of the central nervous system (CNS) [10,11], are severely affected by the presence of mutated SOD1 and contribute to ALS pathogenesis [12,13,14,15]. A precocious and progressive degeneration of oligodendrocytes, together with alterations in myelin structure, have been found in the ventral GM of the spinal cord of both ALS patients and SOD1^G93A^ mouse model of the disease. Protein aggregates in oligodendrocyte cytoplasm and morphological changes appear before disease onset and prior to MN degeneration [13]. Oligodendroglia loss is accompanied by an impairment in the metabolic support to MNs [11], contributing to their degeneration. In parallel, in the ventral horn region of the spinal cord of SOD1^G93A^ mice [12,13], oligodendrocyte precursor cells (OPCs)—the glial cells able to give rise to mature myelinating oligodendrocytes—increase their proliferation, but this response fails to accomplish full maturation, thus leaving large areas demyelinated [12]. Consequently, an innovative approach to ALS treatment may consist in the implementation of endogenous OPC differentiation in order to restore oligodendrocyte function and prevent motor neuronal degeneration.

Previous studies indicate that the G protein-coupled receptor 17 (GPR17) is a key player in oligodendrocyte differentiation [16,17,18,19]. The receptor is expressed during a specific temporal window of the OPC differentiation process [18] and acts as sensor of damage, since it is abnormally upregulated in many CNS pathologies [16,17,20,21,22]. In particular, fate mapping studies performed in GPR17-iCreER^T2^:CAG-eGreen fluorescent protein (GFP) transgenic mice have shown that the pool of GPR17-expressing OPCs rapidly responds to brain injury by increasing their proliferation rate and migratory capacity, but eventually fails to accomplish terminal maturation [23,24,25]. Importantly, in vitro and in vivo data indicated that abnormal GPR17 upregulation contributes to impaired OPC maturation [26] and myelinogenesis [27], suggesting that, under pathological conditions, prolonged non-physiological over-activation of GPR17 may hamper endogenous reparative processes [28]. Interestingly, in vivo inhibition of GPR17 by non-selective antagonists or antisense oligonucleotides greatly reduces ischemic damage, improves functional recovery and fibers connectivity in rodent models of cerebral ischemia [16,29,30] and promotes remyelination in murine model of lysolecithin-induced demyelination [31]. However, it is still unknown whether GPR17 alterations also occur in the context of ALS pathology, nor is known if GPR17 ligands could prove useful in restoring OPC functions in ALS.

Here, we propose to investigate the expression and the involvement of the GPR17 receptor in the SOD1^G93A^ mouse model of ALS during the progression of the pathology and possibly to validate this purinergic P2Y-like metabotropic receptor as a new potential pharmacological target to correct oligodendroglia dysfunction in ALS [32]. At variance from other targets involved in myelination, GPR17 is a membrane receptor, and therefore amenable for modulation with pharmacological ligands. We first demonstrated that GPR17 expression is strongly upregulated in the spinal cord of SOD1^G93A^ mice at both pre-symptomatic and late stages of ALS pathology. We then showed that OPCs isolated from the spinal cords of SOD1^G93A^ mice displayed no variation in their proliferation capabilities, but an intrinsic defect of differentiation compared to control OPCs. Last, we demonstrated that the in vitro exposure of cultured SOD1^G93A^ OPCs to montelukast, a GPR17 antagonist, can rescue OPC maturation.

## 2. Results

### 2.1. Time-Dependent Upregulation of GPR17 Expression in the Spinal Cord of SOD1^G93A^ Mice

**Figure 1:**
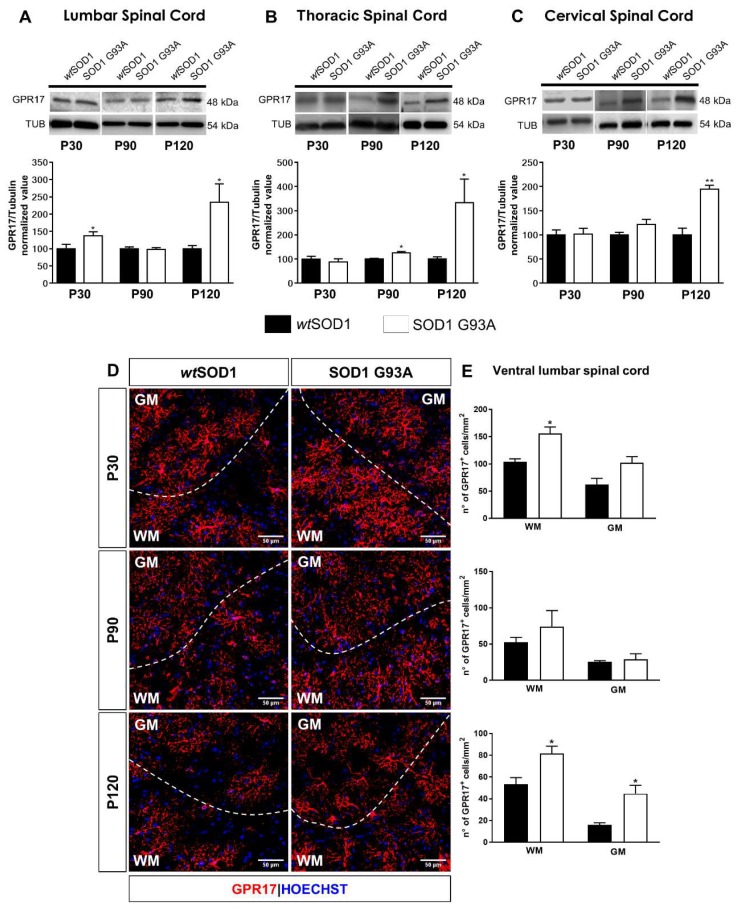
GPR17 expression is increased in spinal cord of SOD1^G93A^ mice. (**A**–**C**) Representative images and quantifications of GPR17 protein levels analyzed by western blot in lumbar (**A**), thoracic (**B**), and cervical (**C**) spinal cord of *wt*SOD1 and SOD1^G93A^ mice at P30, P90, and P120. Histograms show the results of densitometric analysis. Data are expressed as mean ± SE (*n* = 6); * *p* < 0.05; ** *p* < 0.01 SOD1^G93A^ vs. *wt*SOD1, non-parametric Mann–Whitney test. (**D**) Representative images of GPR17 staining in the ventral lumbar spinal cord of *wt*SOD1 and SOD1^G93A^ mice at pre-symptomatic stage P30, early symptomatic stage P90 and late symptomatic stage P120. Dashed line separates white matter (WM) and grey matter (GM). Hoechst 33258 was used to label cell nuclei. Scale bar: 50 µm. (**E**) Histograms show the quantitative analysis of the density of GPR17^+^ cells in WM and in GM of the ventral spinal cord (*n* = 3; 2/3 sections for animal). * *p* < 0.05 SOD1^G93A^ vs *wt*SOD1; Student’s *t*-test.

To characterize disease-related changes of the GPR17 protein, western blot analysis was performed on tissue homogenates obtained from spinal cords of both wild-type SOD1 (*wt*SOD1) and SOD1^G93A^ mice, collected at different ages corresponding to different stages of disease progression (namely, pre-symptomatic stage P30, early symptomatic stage P90 and late symptomatic stage P120). Results indicate that GPR17 protein levels were significantly increased at the pre-symptomatic stage P30, but only in the lumbar spinal cord tract (Figure 1A). Furthermore, a significant increase in GPR17 protein levels was also detected at P90, but only in the thoracic spinal cord tract (Figure 1B). More relevant was the increase observed in all spinal cord tracts of SOD1^G93A^ mice at the late symptomatic stage P120 (Figure 1A–C). To better characterize the pool of GPR17-expressing (GPR17^+^) cells, we performed immunohistochemical analysis focusing on the ventral lumbar spinal cord, that is the region most affected by ALS pathology [33,34,35], of both *wt*SOD1 and SOD1^G93A^ mice, in order to detect possible regional differences between WM and GM areas. As shown in Figure 1D, the majority of GPR17^+^ cells displayed a highly ramified morphology typical of the oligodendroglial lineage and co-expressed the oligodendrocyte transcription factor 2 (Olig2, Figure S1A). The density of GPR17^+^ cells was found to be already significantly increased in the WM of the ventral spinal cord at P30 and, more markedly, in both WM and GM at P120 in SOD1^G93A^ mice with respect to *wt*SOD1 mice (Figure 1D,E). Accordingly, the percentage of Olig2^+^ oligodendroglial cells co-expressing the GPR17 receptor was significantly increased in SOD1^G93A^ mice as compared to *wt*SOD1 animals at both pre-symptomatic and late symptomatic stages (Figure S1B).

**Figure 2:**
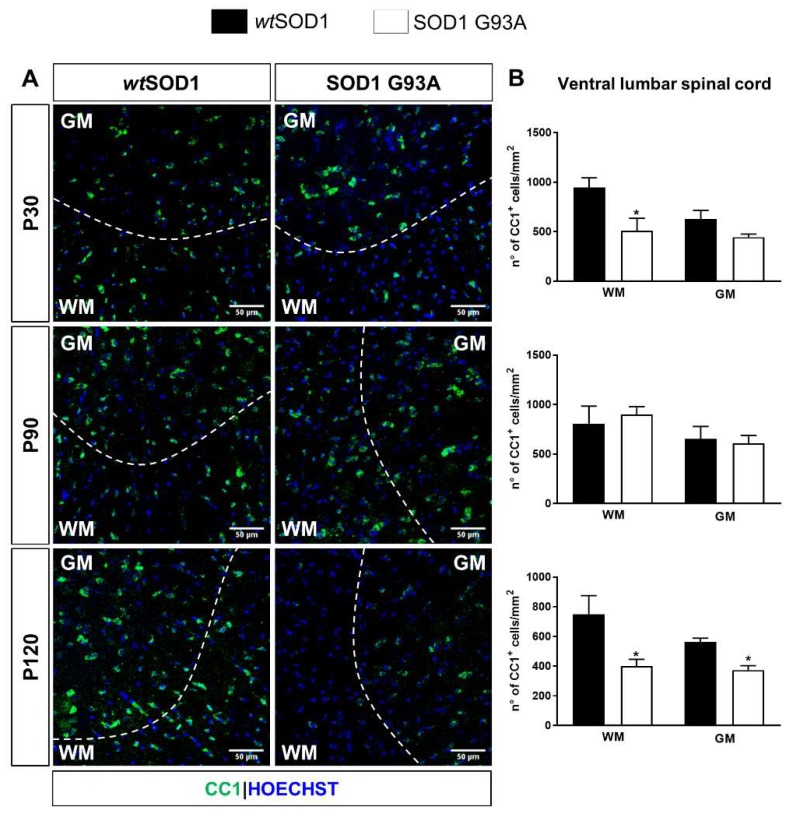
Alterations of mature oligodendroglial cells in lumbar spinal cord of SOD1^G93A^ mice. (**A**) Representative images of CC1 staining in the ventral lumbar spinal cord of *wt*SOD1 and SOD1^G93A^ mice at pre-symptomatic stage P30, early symptomatic stage P90 and late symptomatic stage P120. Hoechst 33258 was used to label cell nuclei. Dashed line separates white matter (WM) and grey matter (GM). Scale bar: 50 µm. (**B**) Histograms show the quantitative analysis of the density of CC1^+^ cells in WM and in GM of the ventral lumbar spinal cord (*n* = 3). * *p* < 0.05, SOD1^G93A^ vs *wt*SOD1; Student’s *t*-test.

In parallel, alterations of the oligodendrocyte differentiation process were assessed by analyzing the density of CC1-expressing (CC1^+^) cells, representing cells that have completed their differentiation process and reached a mature phenotype [36]. The number of mature oligodendrocytes was slightly but significantly reduced at P30 in the ventral lumbar spinal cord WM of SOD1^G93A^ mice. This condition was exacerbated during the late disease progression phase (P120) in the ventral lumbar spinal cord (both in the WM and GM) of SOD1^G93A^ mice compared to *wt*SOD1 mice (Figure 2A,B). A similar reduction at P120 has been observed also for the percentage of Olig2^+^ cells co-expressing CC1 (Figure S1C).

### 2.2. Impaired Oligodendrogenesis in the Developing Spinal Cord of SOD1^G93A^ Mice

**Figure 3:**
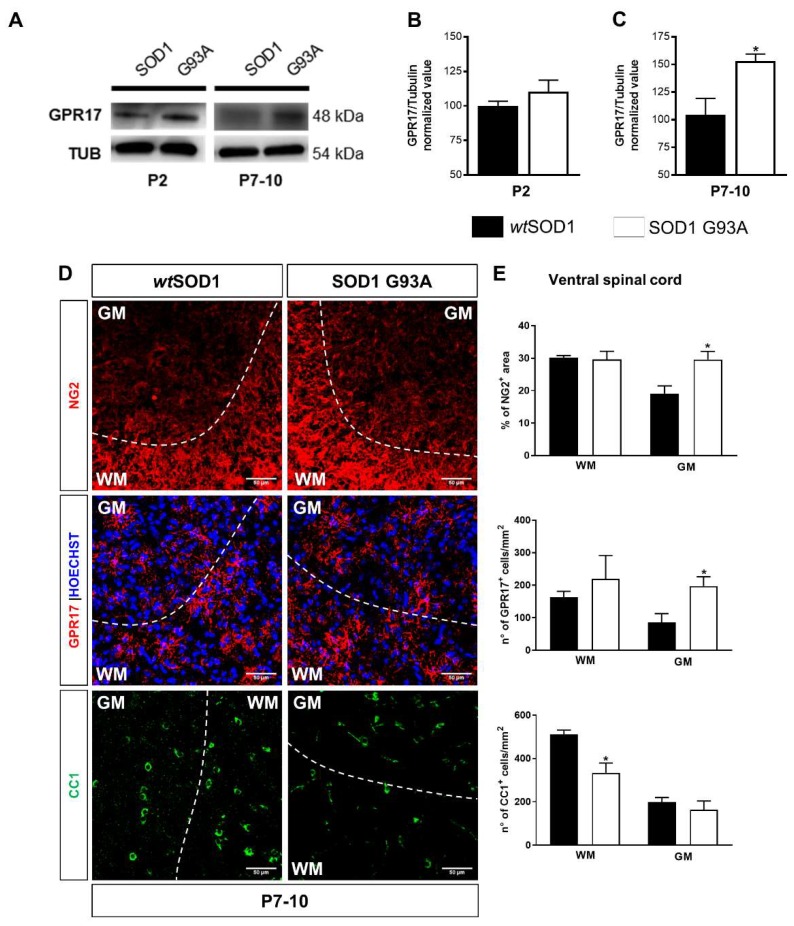
Increase of GPR17 and NG2 expression and impaired oligodendroglial maturation in the developing spinal cord of SOD1^G93A^ mice. (**A**–**C**) Representative images and quantifications of GPR17 protein levels analyzed by western blot in the total spinal cord of *wt*SOD1 and SOD1^G93A^ mice at developmental stages P2 and P7-10. Histograms show the results of densitometric analysis, data are expressed as mean ± SE. (*n* = 7 *wt*SOD1 mice and *n* = 5 SOD1^G93A^ mice); * *p* < 0.05, compared to wtSOD1, non-parametric Mann–Whitney test. (**D**) Representative images of NG2, GPR17, and CC1 staining in the ventral spinal cord of *wt*SOD1 and SOD1^G93A^ mice at P7-10. Dashed line separates white matter (WM) and grey matter (GM). Scale bar: 50 µm. (**E**) Histograms show results of the densitometric analysis of the NG2 staining and of GPR17 and CC1 cell density in WM and in GM of ventral spinal cord (*n* = 3). * *p* < 0.05 SOD1^G93A^ vs *wt*SOD1; Student’s *t*-test.

It is known that, in rodents, myelinogenesis occurs predominantly postnatally within the first 3 weeks [37]. During this process, GPR17 expression progressively increases between P0 and P14 before massive myelin production, occurring around P21 [17]. To assess whether, as already observed for MNs [38], oligodendrocytes may also be altered before ALS symptoms onset, western blot analysis was extended to total spinal cord collected at very early post-natal periods, i.e., P2 and P7-10. Interestingly, a significant increase in GPR17 protein levels was found in the spinal cord of P7-P10 SOD1^G93A^ mice compared to age-matched *wt*SOD1, whereas no statistically significant changes were found at P2 (Figure 3A–C). The increase of GPR17 expression was also confirmed by immunohistochemical analysis which revealed that this upregulation mainly affected the GM of ventral spinal cord of SOD1^G93A^ mice compared to *wt*SOD1 mice (Figure 3D,E). Interestingly, in SOD1^G93A^ mice, we also found an increase of neural glial antigen 2 (NG2) expression in the GM of ventral spinal cord, in parallel with a strong reduction of newborn mature CC1^+^ oligodendrocytes in the WM of ventral spinal cord, suggesting an impairment of the differentiation process in the spinal cord of SOD1^G93A^ mice (Figure 3D,E) during early development.

### 2.3. Impaired Differentiation Capabilities of OPCs Isolated from P7 SOD1^G93A^ Mice

**Figure 4:**
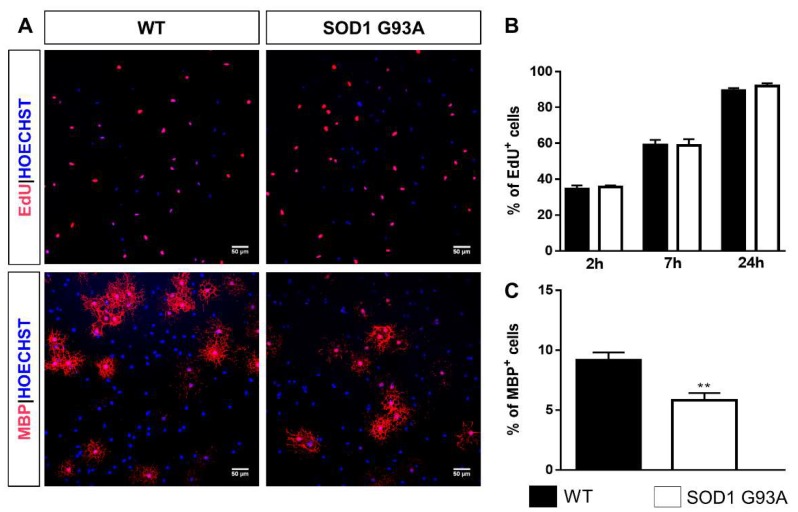
OPCs isolated from P7 SOD1^G93A^ mice exhibit alterations in differentiation capabilities. (**A**) Representative images showing cells from both WT and SOD1^G93A^ OPC cultures that have incorporated EdU in their nuclei or have acquired a mature phenotype (MBP^+^ cells). Hoechst33258 was used to label cell nuclei. Scale bar: 50 µm. (**B**) Histograms showing the quantification of the percentage of proliferating cells after 2, 7, or 24 h treatment with EdU. Data are the mean ± SE of cell counts from at least three coverslips/conditions from three independent experiments. (**C**) Histograms showing the quantification of the percentage of MBP^+^ cells in OPC cultures from WT and SOD1^G93A^ mice. Data are the mean ± SE of cell counts from at least three coverslips/condition from four independent experiments. ** *p* < 0.01 SOD1^G93A^ vs WT; Student’s *t*-test.

**Figure A1:**
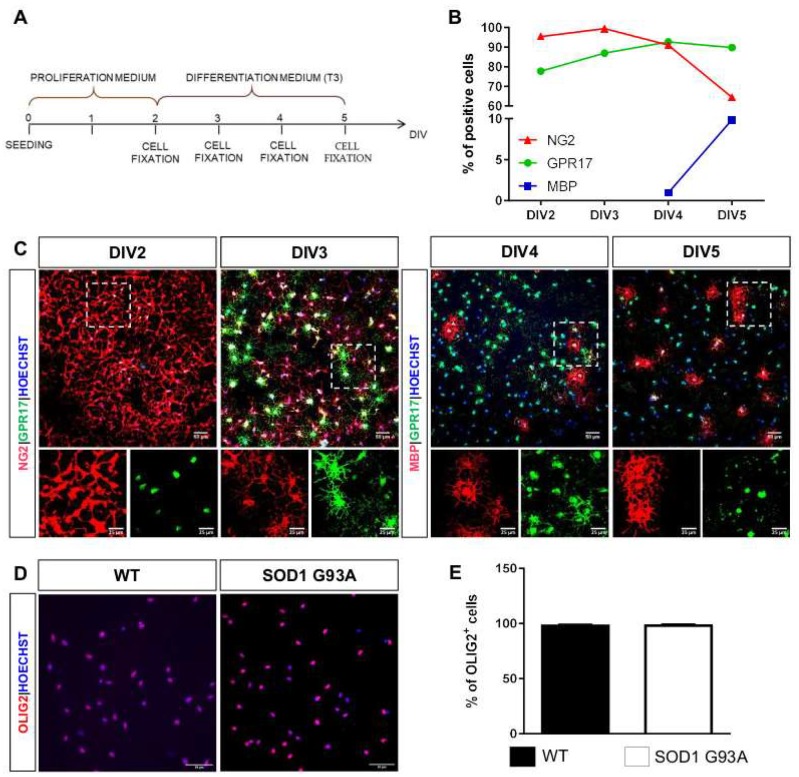
Set up and characterization of primary purified OPCs from spinal cord of P7 wild-type mice. (**A**) Representative experimental protocol scheme for OPC cultures. (**B**) Graph shows the quantification of the percentage of NG2, GPR17 and MBP positive cells at different time points during OPC differentiation. Data are the mean of cell counts from a total of 3 coverslips/condition from seven independent experiments. (**C**) Representative images of cells fixed at DIV2, DIV3, DIV4 and DIV5 showing double immunostaining for GPR17 together with either NG2 or MBP. Hoechst33258 was used to label cell nuclei. Scale bar: 50 μm. Insets show higher magnification of representative GPR17+ cells at different stages of differentiation process, appearing with different morphologies. Scale bar: 25 μm. (**D**) Representative images of OLIG2 staining in primary OPC cultures from spinal cord of WT and SOD1G93A mice. Hoechst33258 was used to label cell nuclei. Scale bar: 50 μm. (**E**) Histograms showing the quantification of the percentage of OLIG2^+^ cells in OPC cultures from WT and SOD1^G93A^ mice. Data are the mean ± SE of cell counts from at least 3 coverslips/condition from 2 independent experiments.

Since, in SOD1^G93A^ mice, GPR17 expression had been found to be already significantly increased in the very early post-natal period, we decided to isolate OPCs from the spinal cord of P7 SOD1^G93A^, *wt*SOD1, and WT mice in order to identify potential alterations in the capacity of these cells to proliferate and differentiate. To perform a detailed characterization of the differentiation process of spinal cord OPCs, cells were fixed after 2, 3, 4, and 5 days in culture (or days in vitro, DIV) as shown in Figure A1A. At the indicated time points, immunocytochemistry analysis was performed to get more information on the morphological characteristics and immunophenotype of GPR17^+^ cells, as described in Appendix A. More than 99% of cells expressed the oligodendroglial marker Olig2, indicating that the OPC culture was pure (Figure A1D,E). The experimental protocol described above was then employed to compare the proliferation rate and differentiation capabilities of OPCs isolated from spinal cords of P7 SOD1^G93A^, *wt*SOD1, and WT mice (Figure 4A). No significant differences concerning both proliferation and differentiation capabilities were found between WT and *wt*SOD1 mice (Figure S2); thus, OPCs obtained from WT mice were used as control. For proliferation studies, OPCs in proliferation medium were cultured in presence of 5 µM 5-ethynyl-2′-deoxyuridine (EdU) for 2, 7, or 24 h, fixed and stained for EdU. Results indicate no significant variation in the number of cells incorporating EdU in OPCs from SOD1^G93A^ mice compared to OPCs from WT mice (Figure 4B). For the differentiation studies, OPCs were cultured in proliferation medium for 2 days and then in differentiating medium. After 3 days, cells were fixed and stained for GPR17 and myelin basic protein (MBP). A significantly reduced number of OPCs expressing the mature marker MBP (5.33 ± 0.61%) was found in cultures obtained from spinal cords of SOD1^G93A^ mice compared to OPCs from WT mice (9.84 ± 0.74%) (Figure 4C). 

**Figure 5:**
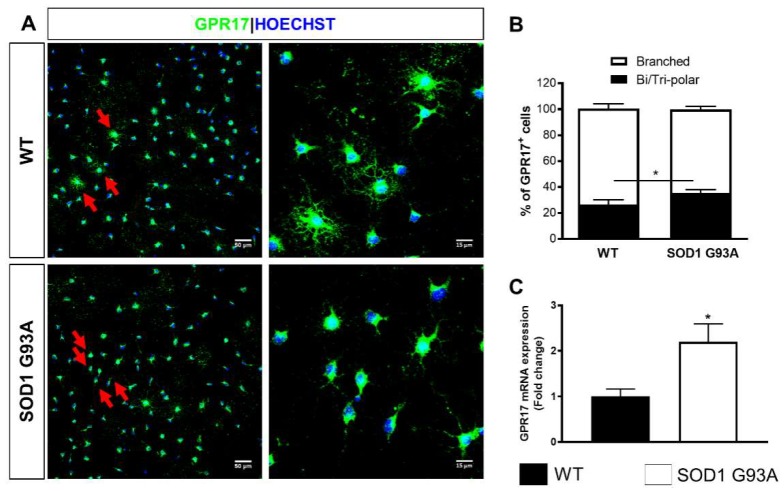
OPCs isolated from P7 SOD1^G93A^ mice are blocked at immature GPR17^+^ stage. (**A**) Representative images showing GPR17^+^ cells in WT and in SOD1^G93A^ OPC cultures. Insets show higher magnification of representative GPR17^+^ cells with branched (in WT cultures) or with bi/tripolar morphology (in SOD1^G93A^ cultures). Arrows indicate GPR17^+^ cells with highly ramified or bipolar/tripolar morphology. Scale bar: 50–15 µm. (**B**) Stacked histograms showing the quantification of the bipolar/tripolar and the branched GPR17^+^ cells. Data are the mean ± SE of cell counts from a total of six coverslips/condition from three experiments. * *p* < 0.05 SOD1^G93A^ vs. WT; Student’s *t*-test. (**C**) Histograms showing GPR17 mRNA level quantification by qRT-PCR in SOD^G93A^ cultures with respect to WT cultures (*n* = 3). * *p* < 0.05 SOD1^G93A^ vs. WT; Student’s *t*-test.

Of note, at this time points, 74.16 ± 3.74% of GPR17^+^ cells of WT cultures exhibited a highly ramified morphology (Figure 5A,B). In SOD1^G93A^ cultures, the number of GPR17-expressing cells with branched processes was significantly reduced to 64.28 ± 2.44% (Figure 5A,B). This result was corroborated by the observation that, at this time point, in SOD1^G93A^ cultures, there still was a higher percentage of GPR17^+^ cells (35.56 ± 2.47%) with simple bi- or tri-polar morphology (Figure 5A,B), confirming that OPCs from SOD1^G93A^ mice do show impaired differentiation capabilities. Interestingly, significantly increased levels of GPR17 mRNA were detected at DIV3 in OPCs from SOD1^G93A^ mice compared to those in OPCs from WT animals (Figure 5C), suggesting that the upregulation of GPR17 could be responsible for the block of OPC differentiation, as already demonstrated for brain OPCs [26].

### 2.4. Restoration of the Differentiation Capabilities of OPCs from P7 SOD1^G93A^ Mice by Montelukast

Data reported above demonstrate that OPCs from SOD1^G93A^ mice exhibit an altered differentiation program in vitro compared to both WT and *wt*SOD1 cells and suggest that a pathological GPR17 upregulation could be at the basis of the detected differentiation block.

**Figure 6:**
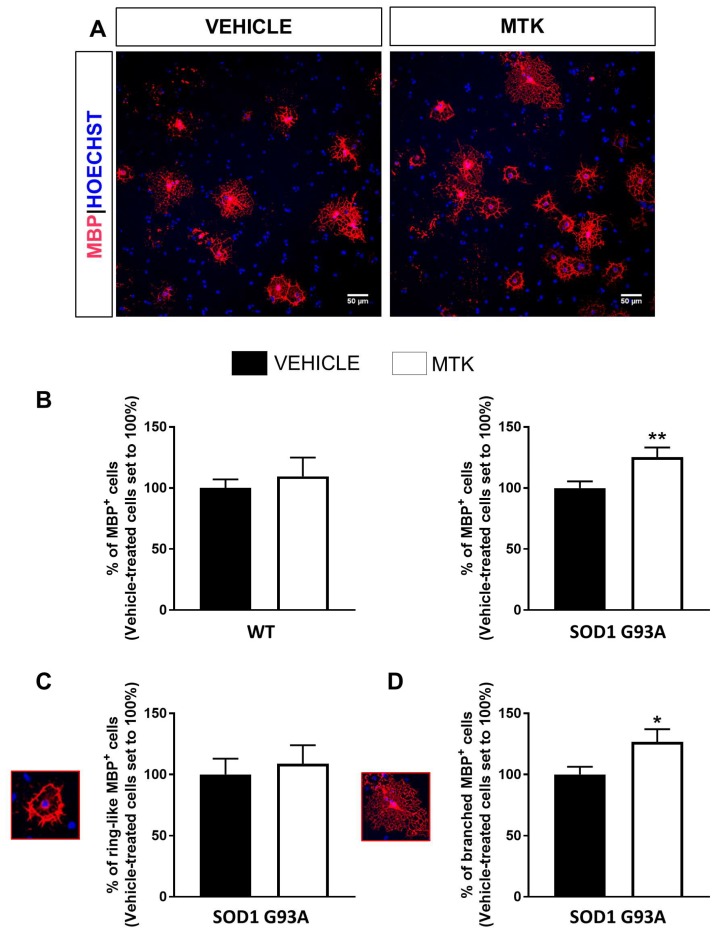
Montelukast increases differentiation in OPCs isolated from spinal cord of SOD1^G93A^ mice. (**A**) Representative images showing MBP^+^ cells in SOD1^G93A^ cultures exposed to montelukast (MTK) or vehicle. Hoechst33258 was used to label cell nuclei. Scale bar: 50 µm. (**B**) Histograms showing the quantification of the percentage of MBP^+^ cells in cultures from WT and SOD1^G93A^ mice exposed to MTK or vehicle (*n* = 6–8 coverslips from three independent experiments). Data are expressed as mean ± SE and vehicle-treated cells have been set to 100%. ** *p* < 0.01 SOD1^G93A^ MTK vs. vehicle, Student’s *t*-test. (**C**) Graph showing the percentage of MBP^+^ cells with ring-like morphology in SOD1^G93A^ cultures exposed to MTK or vehicle (*n* = 7 coverslips form three independent experiments). Data are expressed as mean ± SE and vehicle-treated cells have been set to 100%. (**D**) Graph showing the percentage of MBP^+^ cells with branched morphology in SOD1^G93A^ cultures exposed to MTK or vehicle (*n* = 7 coverslips form three independent experiments). Data are expressed as mean ± SE and vehicle-treated cells have been set to 100%. * *p* < 0.05 MTK vs vehicle, Student’s *t*-test.

To assess whether the pharmacological blockade of GPR17 could restore impaired SOD1^G93A^ OPC terminal maturation, we used the GPR17 antagonist montelukast (MTK) [18,19,39,40] to treat OPCs isolated from the spinal cords of WT and SOD1^G93A^ mice. As shown in Figure 6, MTK was able to significantly increase the percentage of mature MBP^+^ cells in cultured OPCs from SOD1^G93A^ mice, while it had no effect on WT OPC cultures (Figure 6A,B). No effects of the treatment have been detected on either proliferation or cell viability (Figure S3). Importantly, the morphological characterization of cells revealed that the increased number of MBP^+^ cells found after MTK treatment in SOD1^G93A^ cultures could be ascribed to cells displaying a bushy morphology, typical of cells capable of producing myelin sheaths [41], and not to cells with a ring-like structure, which is instead indicative of still immature phenotype (Figure 6C,D). Globally, these results unveil the existence of intrinsic differentiation defects in SOD1^G93A^ OPCs and highlight the GPR17 blockade by its antagonist MTK as an effective approach to correct this dysfunction.

## 3. Discussion

Through the last decades, purinergic signaling has been involved in modulation of OPC proliferation, migration, and myelination. The large number of purinergic receptors identified on both OPCs and mature oligodendrocytes, the different signaling pathways induced by their activation and the combined activity of ectonucleotidase enzymes make this system highly complex [32]. Of note, expression and function of some purinergic receptors were found to be altered under disease conditions characterized by neurodegeneration, aberrant inflammatory response, and oligodendrocyte dysfunction [32]. Moreover, a central role of P1 and P2 receptors in the pathogenesis of ALS has been also suggested [42,43], raising interest for this still unexplored system in view of its exploitation for novel therapeutic opportunities for this incurable disease. Previous studies from our and other laboratories demonstrate that the P2Y-like GPR17 receptor represents a good pharmacological target to implement repair and remyelination under several neurodegenerative conditions including cerebral ischemia, multiple sclerosis, and traumatic brain injury [32,44]. However, so far, the involvement of GPR17 has never been studied in the context of ALS pathology. Understanding whether alterations of GPR17 expression are present in ALS is important to identify the disease stage at which a GPR17-based therapeutic approach would be more relevant. 

Here we show, for the first time, an upregulation in GPR17 expression in the spinal cord of a murine model of ALS. A slight, but significant, increase of GPR17 protein levels was already detectable in the lumbar spinal cord of SOD1^G93A^ mice at pre-symptomatic stage P30 compared to *wt*SOD1 mice. Immunofluorescence analyses showed that GPR17 upregulation occurs specifically in cells belonging to the oligodendrocyte lineage. These results are in line with previous findings reporting oligodendroglial alterations prior to MN degeneration and before symptomatic manifestation of the pathology [13], and the major involvement of the lumbar tract as the region of the spinal cord being most affected by the pathology [33,34,35].

At P90, the difference in GPR17 levels was no longer present in the lumbar tract but was instead slightly detected in the close thoracic tract. This is consistent with the fact that the thoracic and cervical portions are affected by the pathology to a smaller extent, and probably at later stages after clinical onset, compared to the lumbar one. However, the most prominent increase in GPR17 protein expression was detected at late symptomatic stage (P120) in all tracts of the spinal cord. Interestingly, in the lumbar tract, GPR17 upregulation mainly affected the ventral region of spinal cord and was accompanied by loss of mature CC1^+^ oligodendrocytes. These data are in line with reactive changes of OPCs in this area, as already demonstrated previously [45]. 

Altogether, our results confirm that, in a similar way to other experimental models of neurodegenerative disorders, cells expressing GPR17 precociously react to damage also in SOD1^G93A^ mice. Our results also confirm that this reaction is already present at disease pre-symptomatic stage in the lumbar tract. We speculate that, at this early time point, the pool of OPCs expressing GPR17, which is maintained in the adult CNS for repair purposes [23], is rapidly mobilized in order to compensate for initial oligodendroglia dysfunction. This is nevertheless in agreement with already published data indicating that proliferation of NG2^+^ precursors is enhanced at pre-symptomatic stages [12,13]. However, despite this rapid reaction of the GPR17^+^ pool of precursors, these cells fail to properly maturate at late disease stages, as confirmed by the reduction of the number of mature CC1^+^ oligodendrocytes. Recently, mutant SOD1 expression in mature oligodendrocytes has been demonstrated to be sufficient to induce myelin defects driving MN degeneration [15]. On this basis, we speculate that GPR17 upregulation starts very early as a positive event to promote substitution of dysfunctional oligodendrocytes. However, when upregulation is maintained in differentiating OPCs for excessively long times, terminal maturation is impeded and myelination impaired (see also below and [26]). In this respect, several studies have highlighted how GPR17 correct expression timing is the result of the complex integration of intrinsic determinants regulating oligodendroglial differentiation with the extracellular stimuli acting on the *Gpr17* gene [44], that could themselves be altered during ALS disease progression. This important issue still remains to be investigated.

Symptom onset in SOD1^G93A^ mice is a very controversial topic within the scientific community [6,34,46,47,48]. However, although behavioral alterations become evident at adulthood, histological and biochemical modifications mainly affecting MNs are already detectable during embryonic [38] and postnatal development up to P10 [49]. Abnormalities in neuronal architecture, excitability, and axonal transport have been already described at very early stages of the embryonic development [50,51,52]. Interestingly, the first signs of alterations for glial cells, such as activation of astrocyte and microglia and increased number of OPCs have been also described in lumbar spinal cord of SOD1^G93A^ versus *wt*SOD1 mice around P30 [38,48,49,53], but no data are reported before this disease stage. Our immunohistochemical analysis revealed GPR17 upregulation in oligodendrocytes of the ventral spinal cord at very early developmental stages of the disease (P7-10), which is accompanied by a reduction of the number of CC1^+^ mature oligodendrocytes. Globally, the findings of our in vivo studies suggest that, during disease progression, oligodendrocytes exhibit alterations before disease onset in SOD1^G93A^ mice. Importantly, these early defects in oligodendrocytes may be linked to myelin abnormalities that have been previously observed prior to symptoms onset in both zebrafish [15] and murine mutant SOD1 models [54], thus contributing to progressive axonal loss. On this basis, restoring the proper OPC differentiation capability may help to preserve MN functionality and to counteract their degeneration.

To implement the analysis of OPC dysfunction in ALS and to study the effects of pharmacological compounds targeting GPR17, we moved to in vitro studies using primary cultures of spinal cord OPCs from SOD1^G93A^ mice. Results from immunocytochemical analysis showed that, while the proliferation rate is almost the same, the differentiation ability of SOD1^G93A^ OPCs appears to be altered compared to both WT and *wt*SOD1 controls. In fact, after 2 days in proliferation medium and 3 additional days in differentiation medium, the percentage of MBP^+^ cells and the amount of GPR17^+^ OPCs with a more advanced multi-branched morphology were significantly lower in SOD1^G93A^ than in WT mouse cultures. The percentage of GPR17-expressing cells with an immature bi- or tri-polar morphology was instead still high in cultures from SOD1^G93A^ animals compared to WT controls, confirming that mutant cells are less differentiated at this time in culture. Thus, it can be hypothesized that expression of mutated SOD1 in differentiating OPCs may lead to accumulation of reactive species within these cells, which in turn can induce the release of damage signals able to over-stimulate GPR17 expression resulting in a maturation block [44]. These results are in line with previously published data reporting that, in SOD1^G93A^ mice, the newly generated OPCs with repair purposes fail to restore oligodendroglial dysfunction, since they do not reach the final stage of maturation to mature myelinating oligodendrocytes, leaving large areas of demyelination [12]. Very interestingly, our results obtained with spinal cord OPCs are different of those obtained by Ferraiuolo et al., who did not observe any difference in the differentiation capabilities of cortical OPCs obtained from SOD1^G93A^ mice compared to those obtained from WT littermates [14]. A possible explanation of these discrepancies could be found in the different intrinsic properties and heterogeneity of spinal cord and cortical OPCs which are strictly related to their CNS region location [55]. 

Based on previous studies highlighting the involvement of GPR17 in the regulation of OPC differentiation program [18,32], we assessed whether pharmacological targeting of GPR17 could help restoring the maturation program of OPCs from SOD1^G93A^ mice. As detailed above, both our in vivo and in vitro results clearly show that GPR17 is pathologically upregulated within these cells and that this is associated to a concomitant blockade of OPC terminal maturation. Under this condition, we reasoned that the pharmacological manipulation of GPR17 could be useful in re-establishing a correct receptor activity and in restoring OPC functions. In this contest, the use of either an agonist or an antagonist is still a debated issue likely because the conflicting results of in vitro experiments [28,56,57,58,59,60,61,62,63,64]. Previous works had already demonstrated that the in vivo pharmacological inhibition of GPR17 is able to improve WM integrity, increasing the number of mature oligodendrocytes and promoting remyelination [30,31]. Accordingly, our in vitro results demonstrate that MTK, an already marketed drug whose ability to antagonize GPR17 has been previously shown in primary purified OPCs and in the murine Oli-Neu oligodendroglial cell line [18,19] and recently confirmed in radioligand binding studies [40], markedly increased the percentage of cells expressing MBP in comparison to vehicle-treated cells in SOD1^G93A^ cultures. These data confirm that blockade of GPR17 under pathological condition characterized by its abnormal upregulation can successfully induce OPCs to resume their normal differentiation program. Even though this drug is not a selective antagonist of GPR17, its use in primary OPC cultures from SOD1^G93A^ mice—in which CysLT1 receptor mRNA expression was undetectable (as previously described also for WT OPCs [18])—allows us to rule out the observed effects be due to blockade of this cysteinyl leukotrienes receptor subtype. Of course, in vivo administration of MTK in SOD1^G93A^ mice may provide beneficial effects not only by acting on GPR17-expressing OPCs, but also by managing other pathological features of ALS, including oxidative stress and neuroinflammation, as already described for different disease models [30]. Furthermore, being an already marketed orally available drug, MTK may represent a multi-target drug with high translational potential for repurposing strategies [65], thus deserving further evaluations in vivo. 

In conclusion, from this study, GPR17 emerges as a critical player in ALS pathogenesis and a new potential pharmacological target to be exploited to develop novel therapeutic approaches to counteract oligodendrocyte dysfunction in ALS and to retard both MN degeneration and disease progression.

## 4. Materials and Methods 

### 4.1. Animals and Genotyping

B6SJL-TgN SOD1/G93A (+)1Gur mice expressing high copy number of mutant human SOD1 with a Gly^93^Ala substitution (SOD1^G93A^), B6SJL-TgN (SOD1)2Gur mice expressing wild-type human SOD1 (*wt*SOD1) [66], and wild-type (WT) mice were originally obtained from Jackson Laboratories (Bar Harbor, ME, USA) and bred at the animal facility of the Pharmacology and Toxicology Unit, Department of Pharmacy in Genoa. Transgenic animals have been crossed with background-matched B6SJL wild-type female and selective breeding maintained each transgene in the hemizygous state. All transgenic (human SOD1^G93A^ or human *wt*SOD1) and non-transgenic mice (WT) were identified analyzing crude extracts obtained from tail tips. Tail tips were homogenized in phosphate-buffered saline (PBS) solution, lysed by two cycles of freezing and thawing, and centrifuged at 23,000× *g* for 15 min at 4 °C. The SOD1 level was evaluated by staining for its enzymatic activity after 10% non-denaturing polyacrylamide gel electrophoresis [46]. Animals were housed at constant temperature (22 ± 1 °C) and relative humidity (50%) with a regular 12 h/12 h light cycle (light 7:00 a.m.–7:00 p.m.), throughout the experiments. Food (type 4RF21 standard diet obtained from Mucedola, Settimo Milanese, Milan, Italy) and water were freely available. All experiments were carried out in accordance with the European Communities Council (EU Directive 114 2010/63/EU for animal experiments; September 22, 2010), with the Italian D.L. no. 26/2014 and were approved by the local Ethical Committee and by the Italian Ministry of Health (project authorization no. 97/2017-PR). All the animal-involving experiments comply with the ARRIVE guidelines, to minimize animal suffering and to use only the number of animals necessary to produce reliable results. 

### 4.2. Western Blot 

Spinal cords have been collected from SOD1^G93A^ and *wt*SOD1 mice at specific disease stages (i.e., postnatal day—P-2, 7/10, 30, 90, 120) and divided in three different portions (corresponding to cervical, thoracic, and lumbosacral regions). Animal tissues were then lysed and mechanically homogenized in lysis buffer (20 mM Tris pH = 7.2, 0.5% DOC, 1% Triton, 0.1% SDS, 150 mM NaCl, 1 mM EDTA, Sigma Aldrich, Milan, Italy) added with phosphatase inhibitors (2 mM EGTA, Sigma Aldrich) and 1% of protease inhibitors (Sigma Aldrich) for each sample. A range of 25–30 µg aliquots from each protein sample were loaded on 8% sodium-dodecylsulphate polyacrylamide gel and electrophoretically transferred onto PVDF membranes (BioRad Laboratories, Segrate, Italy). Membranes were immunoblotted overnight at 4 °C with rabbit GPR17 antibody (1:300, custom antibody produced by PRIMM, Milan, Italy) and incubated with HRP-conjugated secondary antibodies, both diluted in 5% non-fat dry milk (BioRad Laboratories). Target proteins were detected by ECL detection kit (BioRad Laboratories) and analyzed by ImageJ program. For each sample, α-tubulin (Sigma Aldrich) was used as internal control to normalize GPR17 protein levels and normalized values were used for comparison, expressed as percentage of control lane values as previously described [26].

### 4.3. Immunohistochemistry (IHC) Analysis

At specific disease stages (i.e., postnatal day—P-2, 7/10, 30, 90, 120), SOD1^G93A^ and *wt*SOD1 mice were anesthetized with 100 mg/kg tiletamine/zolazepam and perfused transcardially with PBS (Euroclone, Pero, Italy) followed by 4% neutral buffered formalin (Sigma Aldrich) in deionized water. Spinal cords were collected and post-fixed for 1 h in the same solution at 4 °C, cryoprotected in 30% sucrose for 24 h, embedded in OCT and then frozen at −80 °C. Spinal cords were cut transversally into 20 µm-thick sections with a cryostat and processed for immunofluorescence. Sections were incubated with the following primary antibodies: mouse anti-CC1 (1:100; cat. no. OP80, Millipore, Milan, Italy), rabbit anti-NG2 (1:2000; cat. no. AB5320, Millipore), rabbit anti-GPR17 (1:2500; custom antibody produced by PRIMM, Milan, Italy) and rabbit anti-Olig2 (1:300; cat. no. AB9610, Millipore). Only for GPR17 staining, sections were pre-heated with 10 mM citrate buffer (pH 6) (Sigma Aldrich) containing 0.05% Tween 20 (Sigma Aldrich) for 20 min. Incubation with primary antibodies was made overnight at 4 °C in PBS with 1% normal goat serum (Agilent Technologies Italia, Cernusco sul Naviglio, Italy) and 0.3% Triton X-100. The sections were exposed to the secondary antibodies Alexa 555 and Alexa 488 (Life Technologies, Monza, Italy) for 2 h at room temperature. As to the rabbit anti-GPR17 antibody, signal intensity was enhanced using the High Sensitivity Tyramide-Rhodaminate Signal Amplification kit (Perkin-Elmer, Monza, Italy), following the manufacturer’s instructions. Nuclei were labeled with Hoechst33258 (0.3 μg/mL; Life Technologies). For the quantitative analysis, cells of two or three entire sections of the lumbar spinal cords have been counted using ImageJ. Images were acquired at 20× magnification using a Nikon ECLIPSE Ti2 confocal microscope (Nikon, Florence, Italy). 

### 4.4. OPC Cell Culture

By exploiting our previous expertise in culturing primary rodent OPCs from brain [24,26], we successfully set-up the isolation of OPCs from spinal cord of SOD1^G93A^, *wt*SOD1 and WT mice of postnatal day—P-7. Briefly, spinal cords were dissected from pups at 4 °C and maintained in Tissue Storage Solution^®^ (Miltenyi Biotec, Bologna, Italy) until dissociation; dissected tissues were dissociated into single cell suspensions with Papain-based Neural Tissue Dissociation Kit (Miltenyi Biotec). Platelet derived growth factor α-expressing (PDGFRα^+^) OPCs were then isolated by magnetic activated cell sorting (MACS) separation after incubation with anti-PDGFRα magnetic microbeads (Miltenyi Biotec), following the manufacturer’s instructions. PDGFRα^+^ cells (approximately 60.000 OPCs from each pup) were cultured on poly-d-ornithine (Sigma Aldrich) coated 24-well plates (30.000 cells/well) in OPC medium containing Neurobasal (Life Technologies), 2% B27 (Life Technologies), 1% l-glutamine (Euroclone), 1% penicillin/streptomycin (Euroclone), 10 ng/mL PDGF-AA (Sigma Aldrich), and 10 ng/mL FGF2 (Space Import Export, Milan, Italy). After 2 days, cells were either fixed or switched to oligodendrocyte differentiation medium containing DMEM (Euroclone), 1% N-2 supplement (Life Technologies), 2% B27, 0.01% BSA (Sigma Aldrich), 1% l-glutamine, 1% penicillin/streptomycin, and 10 ng/mL triiodothyronine (T3) (Sigma Aldrich). Cells were differentiated for 3 or 4 days and fixed for immunocytochemistry. Under these culture conditions, contaminating astrocytes and microglia were routinely less than 1% each. 

### 4.5. Immunocytochemistry

Primary OPCs from SOD1^G93A^, *wt*SOD1, and WT mice were fixed at room temperature with 4% paraformaldehyde (Sigma Aldrich) in 0.1 M PBS (Euroclone) containing 0.12 M sucrose (Sigma Aldrich). Labelling was performed incubating cells overnight at 4 °C with the following primary antibodies in Goat Serum Dilution Buffer (GSDB; 450 mM NaCl (Sigma Aldrich), 20 mM sodium phosphate buffer, pH 7.4, 15% goat serum (Life Technologies), 0.3% Triton X-100 (Sigma Aldrich)): rat anti-MBP (1:200; cat. no. MAB386, Millipore, Milan, Italy), rabbit anti-GPR17 (1:50; custom antibody produced by PRIMM, Milan, Italy), rabbit anti-NG2 (1:100; cat. no. AB5320, Millipore) and rabbit anti-Olig2 (1:300; cat. no. AB9610, Millipore). Cells were then incubated for 1 h at RT with the secondary antibody goat anti-rat or goat anti-rabbit conjugated to Alexa Fluor 555 or Alexa Fluor 488 (1:600 in GSDB; Life Technologies). Nuclei were labeled with Hoechst33258. Coverslips were finally mounted with a fluorescent mounting medium (Agilent Technologies) and analyzed using an inverted fluorescence microscope (200M; Zeiss, Milan, Italy) connected to a PC equipped with the Axiovision software (Zeiss). For cell counts, 20 or 40 fields were acquired at 20× magnification (0.07 mm^2^/field; at least three coverslips for each experimental condition). Analysis was performed using the ImageJ software. The results were expressed as a percentage over the number of nuclei, and then normalized versus controls set to 100%. 

### 4.6. Proliferation Assay

After 48 h in culture, SOD1^G93A^, *wt*SOD1, and WT OPCs, maintained in medium containing PDGF-AA and FGF2, were incubated with 5 µM 5-ethynyl-2-deoxyuridine (EdU) for 2, 7, or 24 h at 37 °C and fixed. EdU incorporation was detected with the Click-iT EdU Alexa Fluor-594 Imaging Kit (Life Technologies) according to manufacturer’s protocol. 

### 4.7. Total RNA Extraction, Retrotranscription, and Real-Time PCR

After 1 day in culture in differentiation medium, SOD1^G93A^ and WT cells were lysed with TRIZOL^®^ reagent (Life Technologies). Total RNA was extracted using Direct-zol™ RNA Micro-Prep (Zymo Research, Irvine, CA, USA) according to the manufacturer’s instructions. RNA was then pre-treated with RQ1 DNase (Promega, Milan, Italy) for eliminating genomic DNA contamination. Retrotranscription of 400 ng RNA was performed with SensiFAST™ cDNA synthesis kit (Bioline, London, UK). For real-time PCR, several mixes were prepared according to the number of interested genes. Each mix included Master Mix 2x (Life techonologies), 250 nM probe (for GPR17 Mm02619401_s1, for Rpl13a Mm05910660_g1) and 20 ng of cDNA. Gene-expression was analyzed with TaqMan^®^ Gene Expression Assay and normalized to housekeeping gene Rpl13a expression using CFX96 real-time PCR system (BioRad Laboratories) following the manufacturer’s protocol. 

### 4.8. Pharmacological Treatment

After one day in differentiation medium, primary spinal cord WT and SOD1^G93A^ OPC cultures were treated with the non-selective GPR17 antagonist montelukast (MTK; 1 µM, Cayman, MI, USA) diluted in dimethylsulfoxide (DMSO, Sigma Aldrich). OPCs treated with the same amount of DMSO (indicated as vehicle) alone were used as controls. After 48–72 h, cells were fixed and stained for Olig2 and for the mature cell marker MBP (for details see above). In the same experimental conditions, Olig2^+^ cell viability was evaluated using the Click-iT™ Plus TUNEL Assay Alexa Fluor-488 Kit (Life Technologies) according to manufacturer’s protocol.

### 4.9. Statistical Analisys

All results were expressed as mean ± standard error (SE). Statistical analysis was performed using the nonlinear multipurpose curve-fitting Graph-Pad Prism program (Graph-Pad). The statistical test used was chosen according to the type of experiment performed and was indicated in the legend of the figure. Four degrees of significance were considered: *p* < 0.05 (*), *p* < 0.01 (**), *p* < 0.001 (***), *p* < 0.0001 (****). 

## Supplementary Materials

The following are available online at https://www.mdpi.com/1422-0067/21/7/2395/s1.

## Abbreviations

GPR17	G Protein-coupled Receptor 17
SOD1	Superoxide Dismutase 1
ALS	Amyotrophic Lateral Sclerosis
MN	Motor Neurons
OPCs	Oligodendrocyte Precursor Cells
GM	Grey Matter
WM	White Matter
CNS	Central Nervous System
GFP	Green Fluorescent Protein
WT	Wild Type
OLIG2	Oligodendrocyte transcription factor 2
NG2	Neural Glial antigen 2
MACS	Magnetic Activated Cell Sorting
PDGFRα	Platelet Derived Growth Factor α
T3	Triiodothyronine
MBP	Myelin Basic Protein
DIV	Days In Vitro
EdU	5-Ethynyl-2′-deoxyuridine
MTK	Montelukast
DMSO	Dimethylsulfoxide

## Appendix A

We set up primary purified OPC cultures from spinal cord of P7 wild-type mice. Briefly, dissected spinal cords of P7 WT mice were enzymatically and mechanically dissociated using MACS technology (Miltenyi Biotec; see methods section for details); in order to obtain a pure OPC cell culture, cells expressing PDGFRα (also known as CD140a) were isolated from single-cell suspension, maintained in proliferation medium for 2 days and then switched to a differentiation medium supplemented with T3 for additional 3 days, to promote differentiation into highly ramified MBP-expressing (MBP^+^) cells. The expression of the GPR17 protein in cultured wild-type OPCs was studied in parallel with NG2 and MBP during their spontaneous maturation process (Figure A1). As shown in Figure A1B,C (red line), at DIV2 a 95.43 ± 3.46% of total cell population was positive for NG2 and most cells presented a typical bi-polar morphology with little secondary branching. GPR17 was already detectable in the majority of cells with a bi-polar or tri-polar morphology, even if the protein could be detected as a single intracellular spot close to the nucleus, reminding of localization of the protein into the Golgi apparatus, or also including the initial segments of few processes in tight contiguity with the Golgi apparatus, as previously demonstrated in OPCs isolated from the brain [17]. NG2-GPR17 double-positive cells (NG2^+^/GPR17^+^) accounted for the 80.61 ± 8.02% of the total NG2 population. As OPCs began to differentiate spontaneously in vitro, an increased and strong GPR17 expression was found in NG2^+^ cells with many branched processes emerging from the cell body, reaching a percentage of 93.57 ± 5.72% of NG2^+^/GPR17^+^ cells of the total NG2 population after 1 day in T3 (corresponding to DIV3). The number of GPR17^+^ OPCs continued to increase during differentiation (Figure A1B,C; green line), reaching a maximum peak around DIV4 when they represented about the 97.42 ± 1.1% of the total cell population, and when a significant number of cells acquired a pre-oligodendrocyte phenotype. At this stage, 99% of GPR17^+^ OPCs were still slightly immunoreactive for NG2, whereas only 3.28 ± 0.04% of cells were immuno-positive for MBP. At later stages of maturation, the number of MBP^+^ cells (Figure A1B,C; blue line) progressively increased reaching a 9.87 ± 1.77% at DIV5 with a percentage of GPR17-MBP double-positive cells (GPR17^+^/MBP^+^) of 10.61 ± 2.23%. Nevertheless, MBP^+^ oligodendrocytes that reach terminal differentiation downregulated GPR17, according to previously published data from our laboratory on brain OPCs [18].
