# Supplementary figures and images for: Abnormal Upregulation of GPR17 Receptor Contributes to Oligodendrocyte Dysfunction in SOD1 G93A Mice
Source: Int J Mol Sci. 2020 Mar 31;21(7):2395. doi: 10.3390/ijms21072395 (PMC7177925; doi:10.3390/ijms21072395)

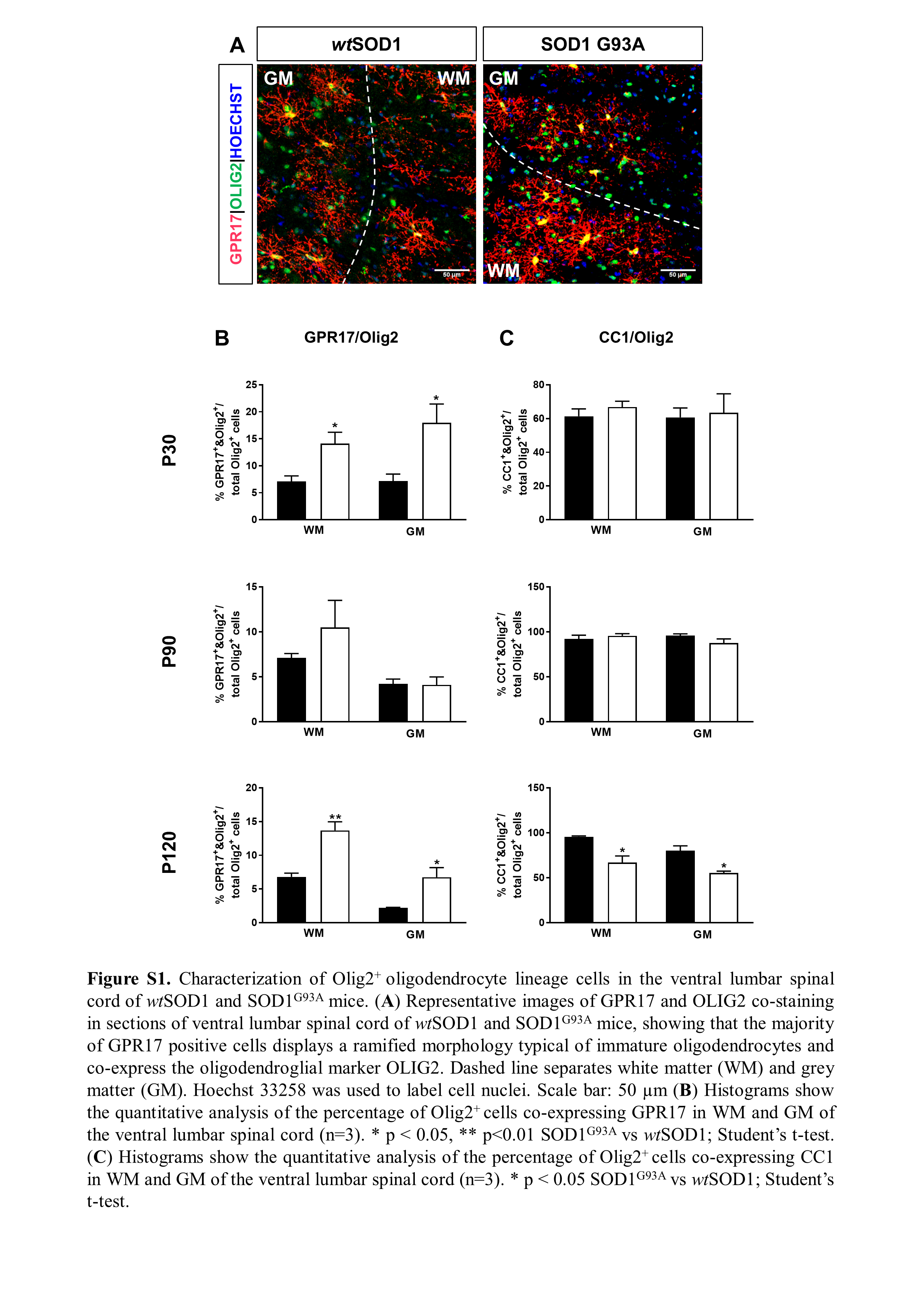

Supplement: Supplementary file 1 [file ijms-21-02395-s001.zip › ijms-721539-Supplementary figures/ijms-721539-Figure S1.tif]

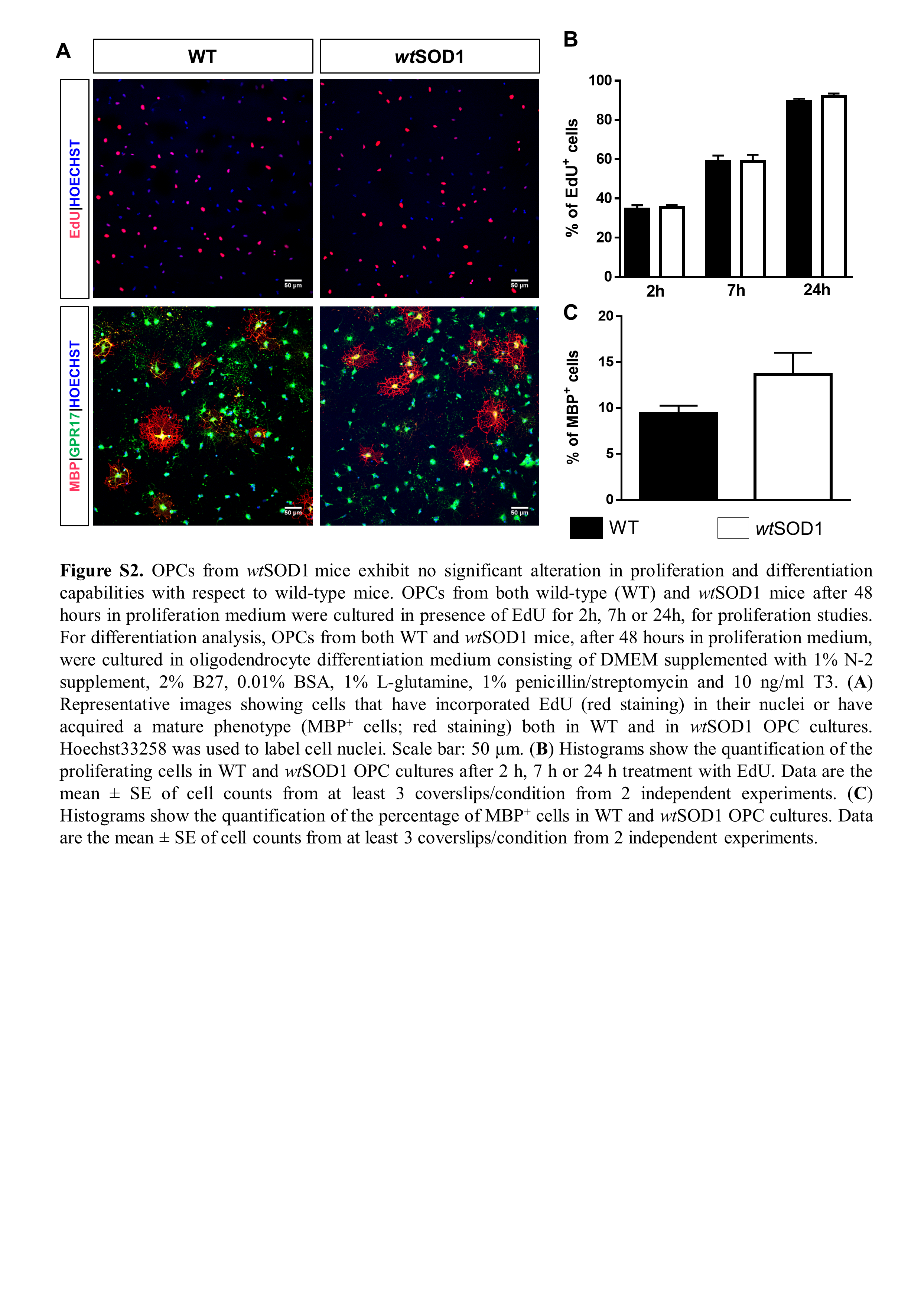

Supplement: Supplementary file 1 [file ijms-21-02395-s001.zip › ijms-721539-Supplementary figures/ijms-721539-Figure S2.tif]

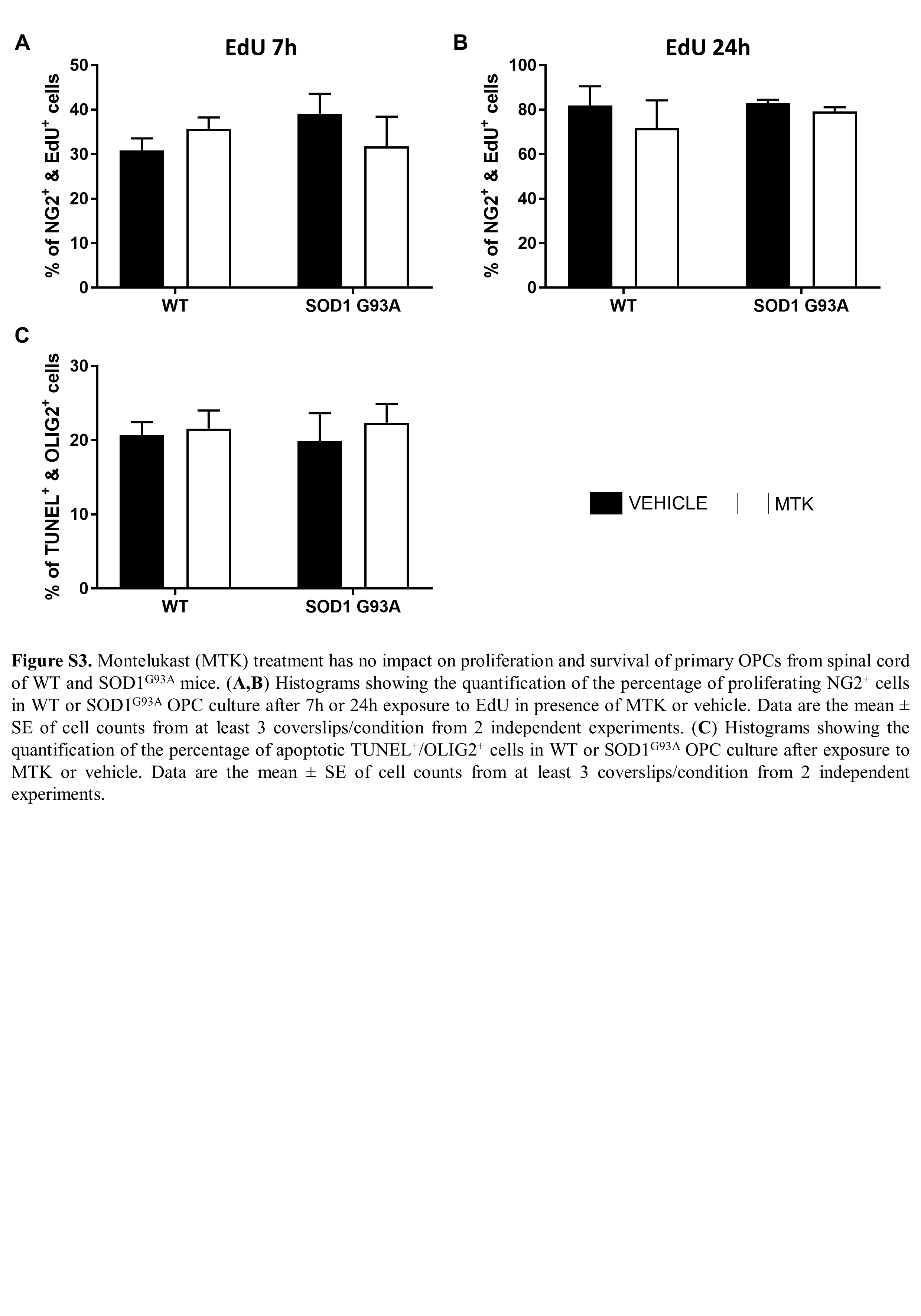

Supplement: Supplementary file 1 [file ijms-21-02395-s001.zip › ijms-721539-Supplementary figures/ijms-721539-Figure S3.tif]
